# Serum miRNAs miR-23a, 206, and 499 as Potential Biomarkers for Skeletal Muscle Atrophy

**DOI:** 10.1155/2017/8361237

**Published:** 2017-10-30

**Authors:** Fei Wang, Jing Wang, Jian He, Wenjiong Li, Jinglong Li, Shengju Chen, Peng Zhang, Hongju Liu, Xiaoping Chen

**Affiliations:** ^1^National Key Laboratory of Human Factors Engineering, China Astronaut Research and Training Center, No. 26 Beiqing Road, Beijing 100094, China; ^2^State Key Laboratory of Space Medicine Fundamentals and Application, China Astronaut Research and Training Center, No. 26 Beiqing Road, Beijing 100094, China

## Abstract

Muscle biopsy has long been expected to be replaced by noninvasive biomarkers with diagnostic value and prognostic applications for muscle atrophy. Growing evidence suggests that circulating microRNAs (miRNAs) could act as biomarkers for numerous pathophysiological statuses. In the present study, our results showed that the serum levels of six muscle-specific miRNAs (miR-1/23a/133/206/208b/499) were all elevated in unloading induced mice. The medium levels of these six muscle-specific miRNAs were all elevated in starvation induced atrophic C2C12 myotubes. Moreover, the serum levels of miR-23a/206/499 were induced in participants after 45 days of head-down bed rest (HDBR). The levels of miR-23a/206/499 were positively correlated with the ratio of soleus volume loss in HDBR participants, indicating that they might represent the process of muscle loss. In conclusion, our results demonstrated that circulating miRNAs could serve as useful biochemical and molecular indicators for muscle atrophy diagnosis and disease progression.

## 1. Introduction

Muscle atrophy is a common physiological and pathological process, which occurs in response to fasting, chronic disease (e.g., cancer, diabetes, AIDS, sepsis, and sarcopenia), and disuse (e.g., long time bed rest and space flight) [1–3]. Skeletal muscle possesses high plasticity controlled by a dynamic balance between protein synthesis and degradation. Increased protein degradation leads to muscle atrophy, whereas increased protein synthesis leads to muscle hypertrophy. Muscle atrophy, induced by increased protein degradation and decreased protein synthesis, leads to the deterioration of disease and reduces the quality of life [4, 5]. Therefore, the diagnosis and treatment of skeletal muscle atrophy is an important clinical issue.

So far, quantification of muscle weight is difficult. Many measurement methods were developed to detect skeletal muscle atrophy, including tomography, magnetic resonance imaging (MRI), and dual-energy X-ray absorptiometry [6]. These methods can detect muscle wasting but cannot indicate the possibility of developing muscle atrophy [7]. Moreover, these methods are all expensive and only available at large institutions. In addition, some potential candidates (e.g., serum creatinine, neoepitope, and collagen type VI fragments) have been tested to use as biomarkers for muscle atrophy [8–10]. But there are always a variety of problems, such as high cost and low accuracy. Thus, it is necessary to discover new noninvasive biomarkers which are cheap and easily available for diagnosis in clinics.

miRNAs are short noncoding RNAs that modulate gene expression on the posttranscriptional level and play key roles in a wide scope of physiological and pathological processes. Some miRNAs are expressed specifically in muscle and named myomiRs [11]. It has been demonstrated that myomiRs play a key role in the proliferation, differentiation, and diseases of skeletal muscle [11, 12]. A number of miRNAs are differentially expressed and are highly involved in the pathophysiological process of denervated muscles [13]. Moreover, it has been reported that miRNAs have been found in a number of body fluids including serum [14]. The profile of serum miRNAs has already been used as biomarker for various diseases, including cancers, heart diseases, and diabetes [14–16]. It has been proved that circulating muscle enriched miRNAs could be used as promising biomarkers for muscle diseases, such as Duchenne Muscular Dystrophy (DMD) diagnosis [17, 18] and Amyotrophic Lateral Sclerosis [19, 20]. However, there was no report about the correlation between serum miRNAs levels and disuse induced muscle atrophy.

The main purpose of this study was to find potential circulating miRNA biomarkers for skeletal muscle atrophy diagnosis. It has been shown that miR-1/23a/206/133/499/208b all play important roles in myogenesis, fiber type determination, or exercise adaptation [21]. We hypothesized that these myomiRs were indicative of muscle atrophy and have potential as biomarkers. We detected serum or medium levels of miRNAs in hindlimb unloaded mice, starved C2C12 myotubes, and HDBR participants. According to our data, we proposed that the serum miRNAs can be used as new biomarkers for muscle atrophy diagnosis.

## 2. Materials and Methods

### 2.1. Animals

In the hindlimb unloading model, we used adult C57 mouse according to previous study [22]. 80 C57 mice (male, 8 weeks old, 20 ± 2 g) were randomly separated into three hindlimb unloading (HU) groups undergoing either 3, 7, or 14 days of hindlimb unloading and one control group was raised for 14 days in normal conditions. All animals were bought from Vital River Laboratories (Beijing, China). All the animal experiments were approved by the Institutional Animal Care and Use Committee of China Astronaut Research and Training Center.

### 2.2. Hindlimb Unloading

All animals were raised at room temperature under 12 h light and 12 h dark, with free access to food and water. Mice were kept in cages one week before experiments for adaptation. The mice hindlimb unloading model has been widely used for studying muscle atrophy [23–25]. Briefly, mice were suspended by strings which were fixed on their tails. The body and floor formed a 45-degree angle to prohibit the hindlimbs from touching the floor or sides of the cage. Control animals were kept on the floor until being euthanized. To maintain the same survival time for each group, animals were grouped as follows: all mice were divided into four groups. HU14 was suspended at the 1st day; HU7 was suspended at the 8th day; HU3 was suspended at the 12th day. All mice were sacrificed at end of HU by cervical dislocation. The soleus, gastrocnemius, and plantaris muscles from both sides of the mice hindlimbs were immediately removed, weighed, and frozen in liquid nitrogen.

### 2.3. Histological Analysis

The histological analysis was performed as described previously [26]. Briefly, the soleus muscles were dissected from the hindlimb of mice. The dissected muscles were frozen using OTC (Thermo, USA) in dry ice cold isopentane. The frozen soleus muscles were sliced into 15 *μ*m cross sections using cryotome (Thermo, USA). The frozen sections were then permeabilized using 0.2% Triton X-100 for 30 min at room temperature, washed by PBS for 3 times, and blocked by 5% goat serum in PBS for 1 h. The frozen sections were immunostained by mouse anti-laminin (dilution: 1 : 300; sc-133241, Santa Cruz Biotechnology). And the second antibody was Alexa Fluor® 594 Goat Anti-Mouse IgG (H + L) (dilution: 1 : 500; A-11005, Invitrogen). The average of the cross-sectional areas was calculated from 300 fibers in each section by Image-Pro Plus 6.0 software (Media Cybernetics Corporation).

### 2.4. Cell Culture, Differentiation, and Starvation

C2C12 myoblasts (gift from Dr. Haitao Wu, AMMS, China) were cultured using DMEM (Gibco, Grand Island, NY) containing 10% fetal bovine serum (Gibco, Grand Island, NY) and 1% penicillin and streptomycin (Gibco, Grand Island, NY) at 37°C and 5% CO2. Myoblasts were induced to differentiation by differentiation medium (DM): DMEM with 2% horse serum (Gibco, Grand Island, NY) and 1% penicillin and streptomycin (Gibco, Grand Island, NY). Myotubes were cultured by DM for 5 days and the DM were replaced every 24 h. Myotube atrophy was induced by serum-depriving. The mature myotube was treated with PBS containing Ca2+ and Mg2+ instead of DM for 4 hours before culture medium (2 ml) was harvested. The myotube starvation model was performed according to previous study [22].

Myotube cultures were photographed under a phase contrast microscope at 400x magnification before and after serum-depriving treatment. Myotube diameters were quantified by measuring a total of >100 tubes diameters from 10 random fields at 100x magnification using Image-Pro Plus 6.0 as described [27].

### 2.5. Subjects and HDBR Protocol

Healthy adult males were used in the HDBR experiment according to previous study [28]. Fifteen healthy male participants aged 27.42 ± 3.89 and 172.65 ± 3.4 cm in height and 64.5 ± 6.7 kg in weight participated in this experiment. The participants were free of diseases and were not athletes. In the process of HDBR, subjects lay in bed and formed a −6° angle with the horizontal ground. The room temperature was maintained at 25 ± 0.5°C and the relative humidity was kept at 60–70%. Subjects taken the head-down position for 24 h per day, with all activities performed in bed. Sterile peripheral blood samples were gained from the eleven participants 1 day before and during (R15, R30, and R45) the HDBR at 6:00 a.m. The soleus volume, before and after 45 days of HDBR, was measured by MRI. Serum was obtained by centrifugation from blood samples and frozen at −80°C. The experiments were approved by the Ethics Committee of China Astronaut Research and Training Center. The participants were informed about the risks and the details before the experiment. The written consent was obtained.

### 2.6. RNA Isolation and Real-Time PCR

In mice, blood samples were collected from the eyes. In HDBR participants, serum samples were prepared from 2 ml of blood taken in BD vacutainer tubes. The blood sample was allowed to clot at room temperature for 30 minutes, followed by centrifugation at 3000*g* for 10 minutes. Cell culture medium samples were centrifuged at 12000*g* for 10 minutes. Serum and cell culture medium supernatant were carefully collected and stored at −80°C until use.

The RNA isolation and real-time PCR analysis were performed as described previously [26]. Briefly, RNA was extracted from serum or cell culture medium by TRIzol LS reagent according to the manufacturer's protocols (Invitrogen, NY). For each sample, 0.5 *μ*g of RNA was reverse transcribed by RevertAid First Strand cDNA Synthesis Kit (Fermentas, NY) as described previously [26]. Real-time PCR was performed by Power SYBR® Green (Applied Biosystems, NY) using Step-one Plus (ABI, NY) for at least three times per experiment. The data were analyzed using the comparative Ct method and normalized by cel-mir-39 which was added to the sample according to the volume. For the real-time PCR measurement of mature miR-1/23a/133/206/208b/499 expression, we used the commercial reverse transcription and PCR primer kit (RiboBio, Guangzhou, China).

### 2.7. Statistical Analysis

Comparisons among multiple groups were performed using one-way ANOVA, followed by unpaired Student's *t*-tests. Comparisons between two groups were performed using unpaired Student's *t*-test; *p* < 0.05 was taken as significant difference.

## 3. Results

### 3.1. Change in Muscle Weight and Cross Section Area following Hindlimb Unloading (HU)

**Figure 1:**
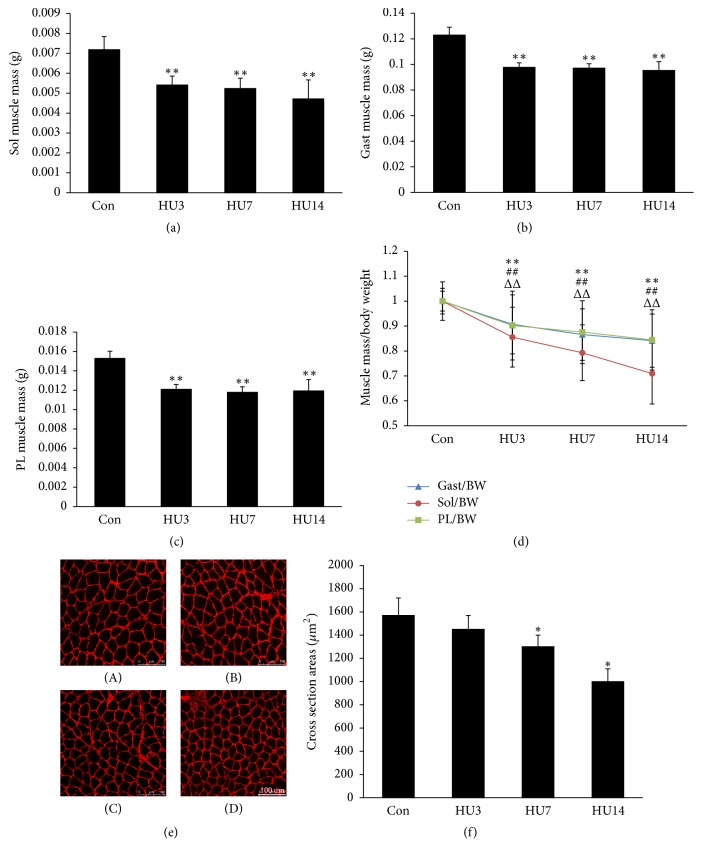
*Change in muscle weight and cross section areas following HU*. (a–c) The hindlimb skeletal muscles (soleus, gastrocnemius, and plantaris) were weighed during 14 days (0, 3, 7, and 14 days) of hindlimb unloading. Data were showed with mean ± SEM (*n* = 20 for each group, ^*∗∗*^*p* < 0.01 versus Con). (d) The relative weight of hindlimb skeletal muscles (gastrocnemius, soleus, and plantaris) was shown during 14 days (0, 3, 7, and 14 days) of hindlimb unloading. The value of muscle/body weight ratio of Con was taken as 1 in each determination. Data were showed with mean ± SEM (*n* = 20 for each group, ^*∗∗*^*p* < 0.01, ^##^*p* < 0.01, and ^ΔΔ^*p* < 0.01 versus its own Con). (e) Representative images of immunofluorescence staining of soleus muscles in Con and hindlimb unloading (3, 7, and 14 days) group using anti-laminin (red) antibody. Scale bar: 100 um (*n* = 5 for each group). (f) Cross section areas of soleus muscles in Con and HU (3, 7, 14 days) group. Data were showed with mean ± SEM (*n* = 5 for each group, total fibers in each muscle cross section were measured, ^*∗*^*p* < 0.05 versus Con). Con, control; HU3, hindlimb unloading for 3 days; HU7, hindlimb unloading for 7 days; HU14, hindlimb unloading for 14 days.

The soleus, gastrocnemius, and plantaris muscles were dissected from both sides of mice hindlimbs. The extracted muscles were weighed. The weight of soleus, gastrocnemius, and plantaris muscle at different time of HU was shown in Figures 1(a), 1(b) and 1(c), respectively. The muscle weight decreased rapidly after 3 days of HU and lost 37%, 20%, and 25% following 14 days of HU in soleus, gastrocnemius, and plantaris muscle, respectively, compared with the control group. Furthermore, muscle weight was adjusted to body weight and presented relative to the control. The ratio of muscle/body weight also reduced significantly following HU (Figure 1(d)). To further evaluate HU-induced atrophy, frozen sections of soleus muscle from the control and unloaded mice were immunostained with anti-laminin (Figure 1(e)). The fiber cross-sectional areas were measured. The mean fiber cross-sectional areas (CSAs) of the soleus muscle declined gradually following 14 days of HU (Figure 1(f)). The results indicated that 14 days of hindlimb unloading induced muscle atrophy in soleus, gastrocnemius, and plantaris muscle, with the greatest extent of atrophy being observed in the soleus.

### 3.2. Serum miRNAs Levels in Mice following HU

**Figure 2:**
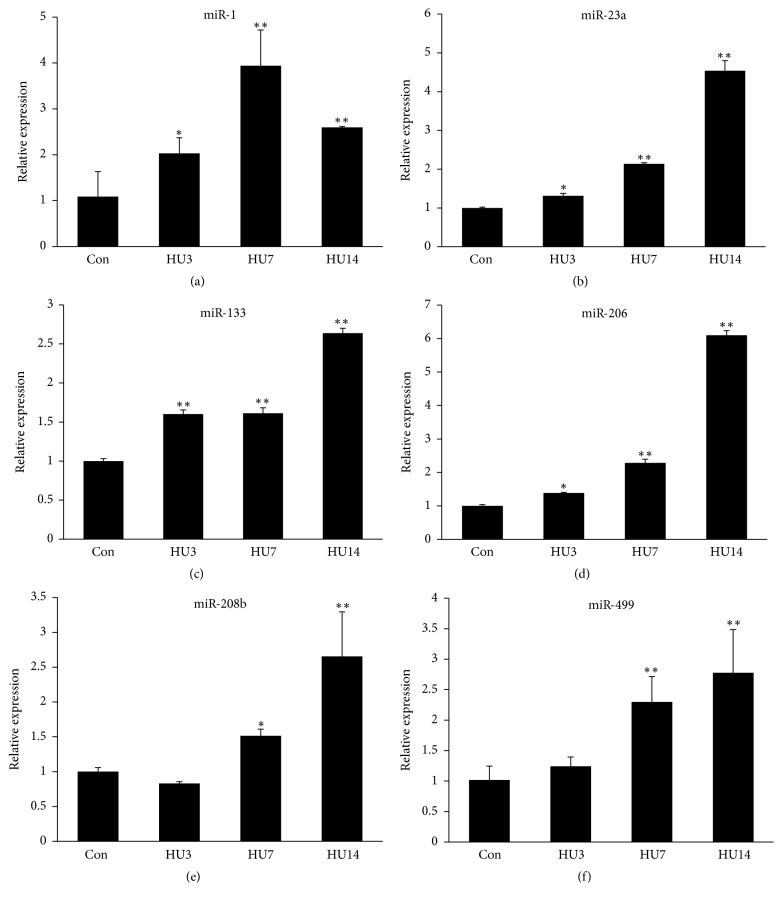
*Serum miRNAs levels in mice following HU*. (a–f) Serum levels of miR-1/23a/133/206/208b/499 in HU-induced atrophic mice and controls (*n* = 20 for each group) were determined by real-time PCR. ANOVA was used for statistical analysis. ^*∗*^*p* < 0.05; ^*∗∗*^*p* < 0.01.

To evaluate the value of serum miRNAs as potential biomarkers for muscle atrophy diagnosis, we detected the serum levels of miR-1/23a/133/206/208b/499 in hindlimb unloaded mice by real-time PCR. Our results showed that the serum levels of miR-1/23a/133/206/208b/499 were all significantly induced (Figure 2). The level of miR-1 declined at HU14 compared with HU7, which may be due to reduced expression or secretion of miR-1 by muscle cells. Moreover, the levels of miR-23a, miR-206, and miR-499 increased during HU in a time-dependent manner. This indicated that the serum levels of myomiRs (miR-1/23a/133/206/208b/499) could be used as biomarkers of muscle atrophy.

### 3.3. Medium miRNAs Levels of C2C12 Myotubes following Starvation

**Figure 3:**
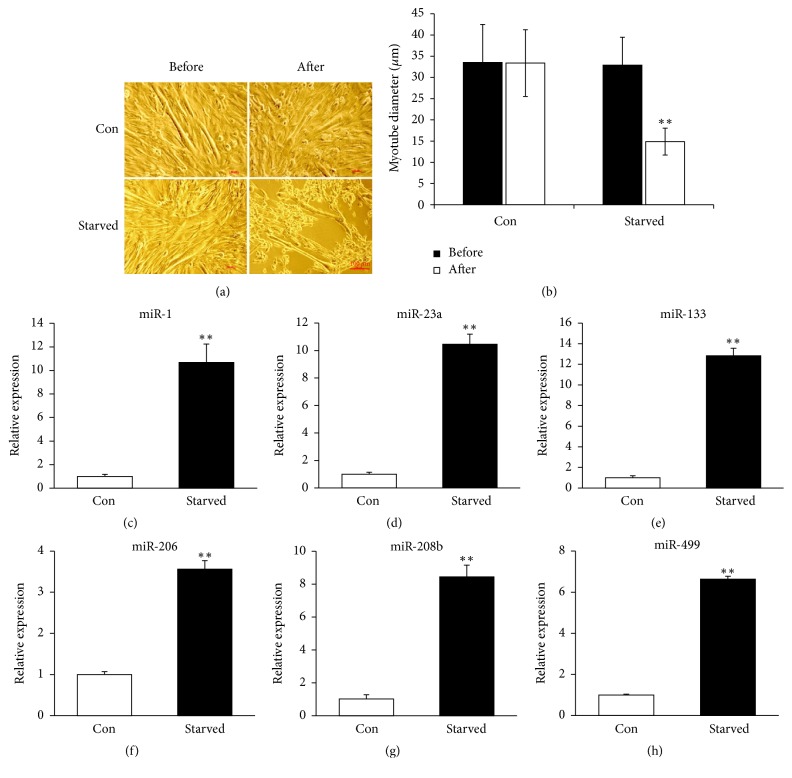
*Medium miRNAs levels of C2C12 myotubes following starvation*. (a) Phase images of C2C12 myotubes before and after starvation. (b) Quantitative analysis of C2C12 myotube diameters before and after starvation. (c–h) Medium levels of miR-1/23a/133/206/208b/499 in starvation induced atrophic C2C12 myotubes and controls (Con, *n* = 6; Starved, *n* = 6) were determined by real-time PCR. *t*-test was used for statistical analysis. ^*∗∗*^*p* < 0.01. Con, control; Starved, atrophic C2C12 myotubes induced by starvation.

To verify the effects of muscle atrophy on the secretion of these six myomiRs, we used starvation model of C2C12 myotubes to induce muscle atrophy* in vitro*. The C2C12 cells were induced to form mature myotubes by differentiation medium for 5 days and were treated with PBS for 4 hours to induce atrophy. 4 hours of starvation led to severe atrophy of C2C12 myotubes (Figure 3(a)), and the diameters of myotubes were reduced by 56% compared with the control (Figure 3(b)). The protein expression level of Atrogin-1 was significantly induced after starvation (see Supplementary Figure 1 in Supplementary Material available online at https://doi.org/10.1155/2017/8361237). The medium levels of miR-1/23a/133/206/208b/499 were all significantly induced, which was consistent with the results* in vivo*. Our results indicated that starvation induced C2C12 myotubes atrophy led to the secretion of miR-1, miR-23a, miR-133, miR-206, miR-208b, and miR-499 into the culture medium, which could be used as indicators for muscle atrophy.

### 3.4. Serum miRNAs Levels in Subjects following HDBR

**Figure 4:**
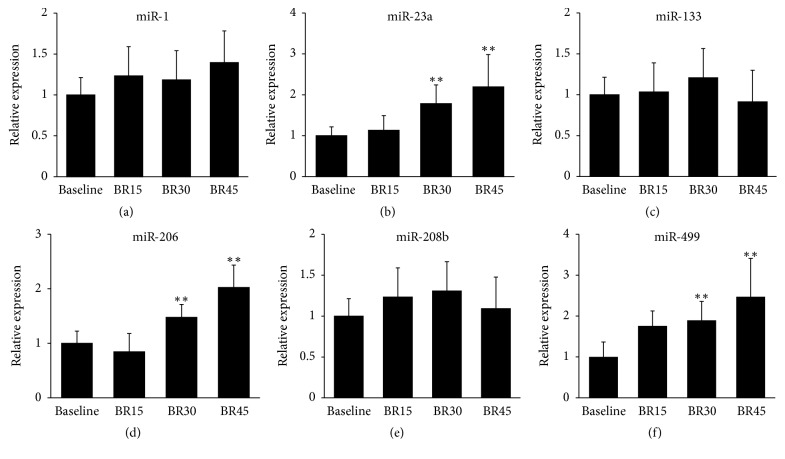
*Serum miRNAs levels in subjects following HDBR*. (a–f) Serum levels of miR-1/23a/133/206/208b/499 in participants before and during HDBR were determined by real-time PCR. ANOVA was used for statistical analysis. ^*∗∗*^*p* < 0.01. BR15, head-down bed rest for 15 days; BR30, head-down bed rest for 30 days; BR45, head-down bed rest for 45 days.

45 days of HDBR resulted in muscle atrophy in the soleus muscle of subjects. The volume of soleus muscle detected by MRI was reduced after 45 days of HDBR (Supplementary Table 1). And the serum levels of miR-1/23a/133/206/208b/499 were detected before and after 45 days of HDBR. The results show that the levels of miR-23a, miR-206, and miR-499 were induced after 30 and 45 days of HDBR (Figures 4(b), 4(d), and 4(f)). However, no significant changes were found in the levels of miR-1, miR-133, and miR-208b following HDBR (Figures 4(a), 4(c), and 4(e)).

### 3.5. The Correlation between Soleus Volume Loss and Serum miRNA Level

**Figure 5:**
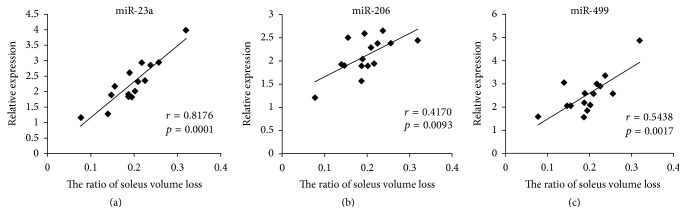
*The correlation between soleus volume loss and serum miRNA levels*. (a–c) The correlation between miR-23a (a), miR-206 (b), miR-499 (c) levels, and the ratio of soleus volume loss in 15 HDBR participants. Regression lines are displayed. *r*, correlation coefficient.

We hypothesize that the change of serum miRNA levels might correlate with muscle loss during HDBR. To verify the hypothesis, a case-by-case correlation assay was performed between the ratio of soleus volume loss and serum miRNA levels of subjects after 45 days of HDBR. As expected, serum miR-23a, miR-206, and miR-499 levels were positively correlated with the ratio of soleus volume loss in subjects after 45 days of HDBR (Figure 5). The correlation of the other three miRNAs was also provided in Supplementary Figure 2. These results indicated that serum levels of miR-23a, miR-206, and miR-499 might represent the extent of muscle degeneration following HDBR.

## 4. Discussion

The change of loading status leads to drastic morphological and functional adjustments to antigravity muscles. Particularly, the antigravity soleus muscle displays dramatic muscle atrophy during unloading [29, 30]. Although medical image system such as MRI could be used to detect muscle atrophy, it is expensive and cannot indicate the risk of developing muscle atrophy. The discovery of serum biomarkers can be useful to diagnose and prognosis muscle atrophy. A growing body of evidence has suggested that circulating miRNAs were potential biomarkers for muscle diseases, like Duchenne Muscular Dystrophy (DMD) and Amyotrophic Lateral Sclerosis (ALS). But there is no study which focused on the relationship between serum miRNAs levels and muscle atrophy. Our results indicated that serum miRNAs could act as potential biomarkers for muscle atrophy diagnosis.

In this study, we used hindlimb unloading model to induce muscle atrophy. This model was widely used to induce muscle atrophy [25, 31]. After 14 days of unloading, the wet weights of soleus, gastrocnemius, and plantaris muscles were all reduced, with the greatest extent of muscle atrophy being observed in soleus muscle. Compared with the control group, the soleus muscle weight and the ratio of muscle/body weight decreased 37% and 29% after 14 days of HU, respectively. Furthermore, we detected the CSAs of soleus muscle, which declined following hindlimb unloading in a time-dependent manner. This data indicated that 14 days of HU could lead to severe muscle atrophy in hindlimb muscles, especially in soleus muscle. The elevations of serum myomiRs (miR-1/23a/133/206/208b/499) were discovered. As these six miRNAs were skeletal muscle enriched miRNAs, we next verified the myomiRs profiles* in vitro* by the C2C12 myotubes starvation model. The levels of miR-1/23a/133/206/208b/499 were all elevated in the medium of C2C12 myotubes after starvation induced atrophy. High-intensity exercise also caused increased levels of miR-1, miR-133, and miR-206 in the plasma [32]. So, the increased levels of miR-1, miR-133, and miR-206 may be at least in part due to the stress caused by the unloading condition.

These myomiRs all play important roles in skeletal muscle. Recent studies showed that both the miR-1/miR-206 family and miR-133 family of miRNAs were upregulated in myocytes during differentiation, but their effects on myogenesis were different. It has been reported that the miR-1/206 family promotes myogenesis; however miR-133 inhibits myogenic differentiation and sustains myoblast proliferation [33]. Wada et al. reported that miR-23a represses the expression of both atrogin-1 and MuRF1 by binding to its 3′UTR [34]. The overexpression of miR-23a could repress muscles atrophy both* in vitro* and* in vivo*. Moreover, Hudson et al. demonstrated that, during glucocorticoid-induced atrophy, Dex treatment induces the secretion of exosomes from C2C12 myotubes into the medium, enriched with miR-23a and miR-1 [35]. Their results suggest that Dex can induce the secretion of miR-23a by exosomes. miR-208b and miR-499 were reported to control muscle fiber type by inhibiting fast muscle-specific genes while promoting slow myofiber genes. In the soleus muscles of humans and rats, spaceflight and hindlimb unloading were known to reduce slow myosin heavy chain and induce fast myosin heavy chain [36, 37].

As it is difficult to perform human physiology in-flight, some ground-based models are used to mimic spaceflight adjustments. The −6° head-down bed rest is identified as the best model to mimic multisystem responses to microgravity by NASA [38]. Several studies of muscle weight change have been performed by this model [39, 40]. Some of these studies showed that the change of muscle weight mimics the adjustment found in astronauts under microgravity. In our research, significant increases were found in the serum levels of miR-23a/206/499 during the HDBR. The levels of miR-23a/206/499 were positively correlated with the ratio of soleus volume loss in HDBR subjects. These results were easy to understand; as atrogin-1 and MuRF1 are the target genes of miR-23a, the secretion of miR-23a by exosome should facilitate the induced expression of these atrophy-inducing genes. miR-206 promoted myogenesis, which was suppressed during muscle atrophy. As miR-499 repressed fast muscle-specific genes while activating slow myofiber genes, the secretion of miR-499 should facilitate the slow to fast myofiber type transition. As the levels of miR-23a/206/499 were positively correlated with the ratio of muscle loss, they can be used to predict the degree of muscle atrophy. Therefore, our results suggested that the serum levels of miR-23a/206/499 could serve as valuable biomarkers for the diagnosis of muscle atrophy. However, the serum profiles of miR-1, miR-133, and miR-208b were different between human and mice, which required further research.

## 5. Conclusions

There were significantly increased levels of miR-23a, miR-206, and miR-499 in serum of HDBR participants after 45 days of head-down bed rest. These miRNAs were positively correlated with the ratio of soleus volume loss. In conclusion, analysis of miR-23a, miR-206, and miR-499 serum levels proved their potential to serve as powerful noninvasive prognostic biomarkers for muscle atrophy.

## Supplementary Material

Supplementary Figure 1. Atrogin-1 and GAPDH protein levels were analyzed by western blot assay.
Supplementary Figure 2. The correlation between soleus volume loss and serum miRNA levels. (A-C) The correlation between miR-1 (A), miR-133 (B), miR-208b (C) levels and the ratio of soleus volume loss in 11 HDBR participants. Regression lines are displayed. r, correlation coefficient.
Supplementary Table 1. The soleus muscle volume of HDBR participants before and after HDBR.

## Abbreviations

HDBR: Head-down bed rest
MRI: Magnetic resonance imaging
HU: Hindlimb unloading
CSAs: Cross section areas
DMD: Duchenne Muscular Dystrophy
ALS: Amyotrophic Lateral Sclerosis.

## References

[1] Bodine S. C., Baehr L. M. (2014). Skeletal muscle atrophy and the E3 ubiquitin ligases MuRF1 and MAFbx/atrogin-1. *The American Journal of Physiology—Endocrinology and Metabolism*.

[2] Brooks N. E., Myburgh K. H. (2014). Skeletal muscle wasting with disuse atrophy is multi-dimensional: The response and interaction of myonuclei, satellite cells and signaling pathways. *Frontiers in Physiology*.

[3] Schiaffino S., Dyar K. A., Ciciliot S., Blaauw B., Sandri M. (2013). Mechanisms regulating skeletal muscle growth and atrophy. *FEBS Journal*.

[4] Dutt V., Gupta S., Dabur R., Injeti E., Mittal A. (2015). Skeletal muscle atrophy: potential therapeutic agents and their mechanisms of action. *Pharmacological Research*.

[5] Koukourikos K., Tsaloglidou A., Kourkouta L. (2014). Muscle atrophy in intensive care unit patients. *Acta Informatica Medica*.

[6] Heymsfield S. B., Adamek M., Gonzalez M. C., Jia G., Thomas D. M. (2014). Assessing skeletal muscle mass: Historical overview and state of the art. *Journal of Cachexia, Sarcopenia and Muscle*.

[7] Scharf G., Heineke J. (2012). Finding good biomarkers for sarcopenia. *Journal of Cachexia, Sarcopenia and Muscle*.

[8] Patel S. S., Molnar M. Z., Tayek J. A. (2013). Serum creatinine as a marker of muscle mass in chronic kidney disease: Results of a cross-sectional study and review of literature. *Journal of Cachexia, Sarcopenia and Muscle*.

[9] Nedergaard A., Karsdal M. A., Sun S., Henriksen K. (2013). Serological muscle loss biomarkers: An overview of current concepts and future possibilities. *Journal of Cachexia, Sarcopenia and Muscle*.

[10] Nedergaard A., Sun S., Karsdal M. A. (2013). Type VI collagen turnover-related peptides-novel serological biomarkers of muscle mass and anabolic response to loading in young men. *Journal of Cachexia, Sarcopenia and Muscle*.

[11] Kovanda A., Režen T., Rogelj B. (2014). MicroRNA in skeletal muscle development, growth, atrophy, and disease. *Wiley Interdisciplinary Reviews: RNA*.

[12] Wang X. H. (2013). MicroRNA in myogenesis and muscle atrophy. *Current Opinion in Clinical Nutrition and Metabolic Care*.

[13] Li G., Li Q.-S., Li W.-B. (2016). miRNA targeted signaling pathway in the early stage of denervated fast and slow muscle atrophy. *Neural Regeneration Research*.

[14] de Guire V., Robitaille R., Tétreault N. (2013). Circulating miRNAs as sensitive and specific biomarkers for the diagnosis and monitoring of human diseases: promises and challenges. *Clinical Biochemistry*.

[15] Chakraborty C., Das S. (2016). Profiling cell-free and circulating miRNA: a clinical diagnostic tool for different cancers. *Tumor Biology*.

[16] Mirra P., Raciti G. A., Nigro C. (2015). Circulating miRNAs as intercellular messengers, potential biomarkers and therapeutic targets for Type 2 diabetes. *Epigenomics*.

[17] Li X., Li Y., Zhao L. (2014). Circulating muscle-specific miRNAs in Duchenne muscular dystrophy patients. *Molecular Therapy—Nucleic Acids*.

[18] Koutsoulidou A., Kyriakides T. C., Papadimas G. K. (2015). Elevated muscle-specific miRNAs in serum of myotonic dystrophy patients relate to muscle disease progress. *PLoS ONE*.

[19] Cloutier F., Marrero A., O’Connell C., Morin P. (2015). MicroRNAs as potential circulating biomarkers for amyotrophic lateral sclerosis. *Journal of Molecular Neuroscience*.

[20] Tasca E., Pegoraro V., Merico A., Angelini C. (2016). Circulating microRNAs as biomarkers of muscle differentiation and atrophy in ALS. *Clinical Neuropathology*.

[21] Nie M., Deng Z.-L., Liu J., Wang D.-Z. (2015). Noncoding RNAs, emerging regulators of skeletal muscle development and diseases. *BioMed Research International*.

[22] Wang J., Wang F., Zhang P. (2017). PGC-1*α* over-expression suppresses the skeletal muscle atrophy and myofiber-type composition during hindlimb unloading. *Bioscience, Biotechnology and Biochemistry*.

[23] Naito H., Powers S. K., Demirel H. A., Sugiura T., Dodd S. L., Aoki J. (2000). Heat stress attenuates skeletal muscle atrophy in hindlimb-unweighted rats. *Journal of Applied Physiology*.

[24] Lee K., Lee Y. S., Lee M., Yamashita M., Choi I. (2004). Mechanics and fatigability of the rat soleus muscle during early reloading. *Yonsei Medical Journal*.

[25] Morey-Holton E. R., Globus R. K. (2002). Hindlimb unloading rodent model: technical aspects. *Journal of Applied Physiology*.

[26] Wang F., Zhang P., Liu H., Fan M., Chen X. (2015). Proteomic analysis of mouse soleus muscles affected by hindlimb unloading and reloading. *Muscle and Nerve*.

[27] Rommel C., Bodine S. C., Clarke B. A. (2001). Mediation of IGF-1-induced skeletal myotube hypertrophy by Pl(3)K/Alt/mTOR and Pl(3)K/Akt/GSK3 pathways. *Nature Cell Biology*.

[28] Xu X., Tan C., Li P. (2013). Changes of Cytokines during a Spaceflight Analog - a 45-Day Head-Down Bed Rest. *PLoS ONE*.

[29] Ye F., Mathur S., Liu M. (2013). Overexpression of insulin-like growth factor-1 attenuates skeletal muscle damage and accelerates muscle regeneration and functional recovery after disuse. *Experimental Physiology*.

[30] Jayaraman A., Liu M., Ye F., Walter G. A., Vandenborne K. (2013). Regenerative responses in slow- and fast-twitch muscles following moderate contusion spinal cord injury and locomotor training. *European Journal of Applied Physiology*.

[31] Morey-Holton E., Globus R. K., Kaplansky A., Durnova G. (2005). The hindlimb unloading rat model: literature overview, technique update and comparison with space flight data. *Advances in Space Biology and Medicine*.

[32] Cui S. F., Wang C., Yin X. (2016). Similar responses of circulating microRNAs to acute high-intensity interval exercise and vigorous-intensity continuous exercise. *Frontiers in Physiology*.

[33] Chen J. F., Mandel E. M., Thomson J. M. (2006). The role of microRNA-1 and microRNA-133 in skeletal muscle proliferation and differentiation. *Nature Genetics*.

[34] Wada S., Kato Y., Okutsu M. (2011). Translational suppression of atrophic regulators by MicroRNA-23a integrates resistance to skeletal muscle atrophy. *The Journal of Biological Chemistry*.

[35] Hudson M. B., Woodworth-Hobbs M. E., Zheng B. (2014). miR-23a is decreased during muscle atrophy by a mechanism that includes calcineurin signaling and exosome-mediated export. *The American Journal of Physiology—Cell Physiology*.

[36] Caiozzo V. J., Baker M. J., Baldwin K. M. (1998). Novel transitions in MHC isoforms: Separate and combined effects of thyroid hormone and mechanical unloading. *Journal of Applied Physiology*.

[37] Fitts R. H., Riley D. R., Widrick J. J. (2000). Physiology of a microgravity environment invited review: Microgravity and skeletal muscle. *Journal of Applied Physiology*.

[38] Sams C. F., Crucian B. E., Stowe R. P. (2009). Immune status, latent viral reactivation, and stress during long-duration head-down bed rest. *Aviation Space and Environmental Medicine*.

[39] Kamiya A., Iwase S., Kitazawa H., Mano T. (1999). Muscle sympathetic nerve activity (MSNA) after 120 days of 6 degrees head-down bed rest (HDBR). *Environmental Medicine*.

[40] Louisy F., Schroiff P., Güell A. (1997). Changes in leg vein filling and emptying characteristics and leg volumes during long-term head-down bed rest. *Journal of Applied Physiology*.

