# Supplementary material for: Serum miRNAs miR-23a, 206, and 499 as Potential Biomarkers for Skeletal Muscle Atrophy
Source: Biomed Res Int. 2017 Oct 30;2017:8361237. doi: 10.1155/2017/8361237 (PMC5682897; doi:10.1155/2017/8361237)
Supplement: Supplementary file 1 — Supplementary Figure 1. Atrogin-1 and GAPDH protein levels were analyzed by western blot assay. Supplementary Figure 2. The correlation between soleus volume loss and serum miRNA levels. (A-C) The correlation between miR-1 (A), miR-133 (B), miR-208b (C) levels and the ratio of soleus volume loss in 11 HDBR participants. Regression lines are displayed. r, correlation coefficient. Supplementary Table 1. The soleus muscle volume of HDBR participants before and after HDBR. [file 8361237.f1.docx]

Supplementary figure 1.


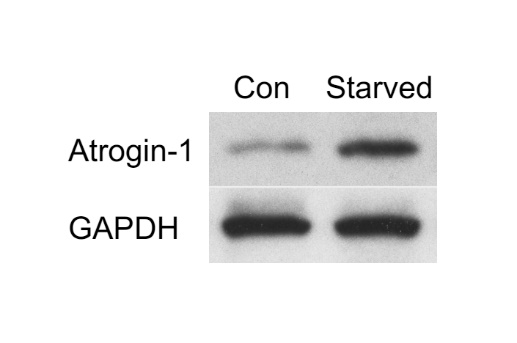


Supplementary figure 2.


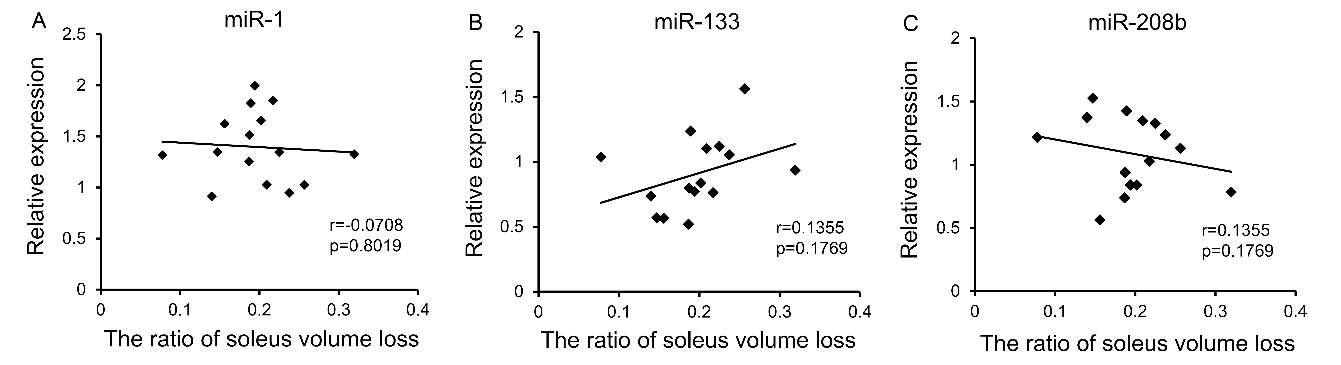


Supplementary table 1.

**Table S1.** The soleus muscle volume of HDBR participants.

|  | baseline (cm3) | |  | HDBR45(cm3) | |
| --- | --- | --- | --- | --- | --- |
|  | left | right |  | left | right |
| A1 | 405.011 | 388.375 |  | 340.33 | 302.975 |
| A2 | 529.532 | 525.759 |  | 416.167 | 409.888 |
| A3 | 323.036 | 334.311 |  | 257.613 | 267.079 |
| A4 | 522.362 | 581.285 |  | 422.504 | 450.306 |
| A5 | 461.121 | 467.079 |  | 386.133 | 368.778 |
| A6 | 494.875 | 461.479 |  | 415.537 | 400.144 |
| A7 | 461.284 | 442.338 |  | 422.51 | 410.858 |
| A8 | 370.73 | 367.977 |  | 307.309 | 288.064 |
| A9 | 520.251 | 519.243 |  | 449.307 | 428.105 |
| A10 | 450.999 | 422.862 |  | 383.346 | 368.36 |
| A11 | 309.097 | 452.639 |  | 251.585 | 266.803 |
| A12 | 414.291 | 409.528 |  | 329.442 | 309.451 |
| A13 | 519.257 | 501.147 |  | 415.235 | 413.614 |
| A14 | 434.551 | 418.457 |  | 313.302 | 320.446 |
| A15 | 392.043 | 371.962 |  | 297.025 | 284.451 |
